# (*E*)-*tert*-Butyl 2-(5-{[4-(dimethylamino)phenyl]diazenyl}-2,6-dioxo-1*H*-pyrimid­in-3-yl)acetate dichloromethane monosolvate

**DOI:** 10.1107/S1600536814007521

**Published:** 2014-04-16

**Authors:** Robert H. E. Hudson, Mohamed E. Moustafa, Paul D. Boyle

**Affiliations:** a 1151 Richmond Street, Department of Chemistry, The University of Western Ontario, London, Ontario, N6A 5B7, Canada

## Abstract

In the title compound, C_18_H_23_N_5_O_4_·CH_2_Cl_2_, the di­chloro­methane solvent mol­ecule is disordered over two sets of sites in a 0.630 (13):0.370 (13) ratio. The dihedral angle between the uracil and phenyl rings is 30.2 (1)°. In the crystal, the principal inter­actions are N—H⋯O hydrogen bonds, which link uracil units across centres of symmetry, forming eight-membered rings with an *R* ^2^_2_(8) graph-set motif. The structure also displays C—H⋯O and C—H⋯Cl hydrogen bonds. Intra­molecular C—H⋯O short contacts are also observed.

## Related literature   

As part of our program in the synthesis of modified nucleobases that possess intrinsic fluorescence while maintaining an unadultered base-paring face, we have prepared an asymmetrical azo compound as a hybrid between a nulceobase and the known fluorescence quencher 4-((4-(di­methyl­amino)­phen­yl)azo)benzoic acid (DABCYL), see: Dodd & Hudson(2009 ▶); Tyagi & Kramer (1996 ▶). For an azo-based fluorescence quencher in peptide nucleic acid, see: Moustafa & Hudson (2011 ▶). For an example of photoisomerization of azo groups in peptide nucleic acid, see: Yue *et al.* (2009 ▶), and in DNA, see: Asanuma *et al.* (1999 ▶). The title compound was prepared following standard procedures, see: Thurber & Townsend (1972 ▶), Tsupak *et al.* (2002 ▶) and Moustafa (2011 ▶). 
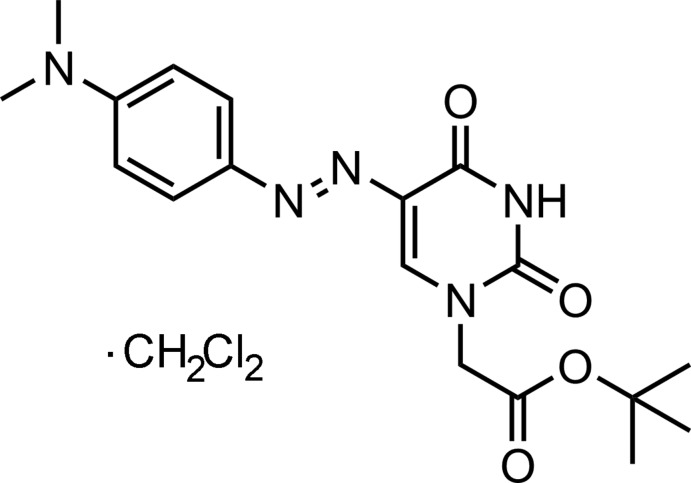


## Experimental   

### 

#### Crystal data  

C_18_H_23_N_5_O_4_·CH_2_Cl_2_
*M* *_r_* = 458.34
Monoclinic, 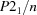

*a* = 13.208 (9) Å
*b* = 10.783 (6) Å
*c* = 17.255 (11) Å
β = 112.33 (2)°
*V* = 2273 (3) Å^3^
*Z* = 4
Mo *K*α radiation
μ = 0.32 mm^−1^
*T* = 150 K
0.20 × 0.18 × 0.15 mm

#### Data collection  

Nonius KappaCCD diffractometer
Absorption correction: multi-scan (*SADABS*; Bruker, 2008 ▶)*T*_min_ = 0.689, *T*_max_ = 0.746
34970 measured reflections
5944 independent reflections
3567 reflections with *I* > 2σ(*I*)
*R*_int_ = 0.054

#### Refinement  

*R*[*F* ^2^ > 2σ(*F* ^2^)] = 0.051
*wR*(*F* ^2^) = 0.128
*S* = 1.03
5944 reflections
374 parameters
12 restraints
H atoms treated by a mixture of independent and constrained refinement
Δρ_max_ = 0.53 e Å^−3^
Δρ_min_ = −0.59 e Å^−3^

### 

Data collection: *COLLECT* (Nonius, 1999 ▶); cell refinement: *SAINT* (Bruker, 2008 ▶); data reduction: *SAINT*; program(s) used to solve structure: *SHELXS2014* (Sheldrick, 2008 ▶); program(s) used to refine structure: *SHELXL2014* (Sheldrick, 2008 ▶); molecular graphics: *NRCVAX* (Gabe *et al.*, 1989 ▶); software used to prepare material for publication: cif2tables.py (Boyle, 2008 ▶). 

## Supplementary Material

Crystal structure: contains datablock(s) I. DOI: 10.1107/S1600536814007521/zp2013sup1.cif

Structure factors: contains datablock(s) I. DOI: 10.1107/S1600536814007521/zp2013Isup2.hkl

Supporting information file. DOI: 10.1107/S1600536814007521/zp2013Isup3.cml

CCDC reference: 995390

Additional supporting information:  crystallographic information; 3D view; checkCIF report

## Figures and Tables

**Table 1:** Hydrogen-bond geometry (Å, °)

*D*—H⋯*A*	*D*—H	H⋯*A*	*D*⋯*A*	*D*—H⋯*A*
N4—H4*A*⋯O2^i^	0.84 (3)	2.02 (3)	2.851 (2)	171 (2)
C2—H2*C*⋯O1^ii^	1.03 (3)	2.62 (3)	3.189 (4)	115 (2)
C4—H4⋯Cl2*X* ^iii^	0.94 (2)	2.98 (2)	3.784 (4)	143.5 (17)
C12—H12⋯O1^iv^	0.99 (2)	2.34 (2)	3.214 (3)	145.8 (18)
C13—H13*B*⋯Cl2*X* ^v^	0.96 (2)	2.99 (2)	3.895 (4)	158.6 (16)
C16—H16*A*⋯O3	0.98 (3)	2.51 (3)	3.042 (3)	114 (2)
C17—H17*C*⋯O3	0.96 (3)	2.48 (3)	2.994 (4)	113 (2)
C1*X*—H1*X*1⋯O3^vi^	0.99	2.52	3.346 (4)	141
C1*X*—H1*X*2⋯O1^ii^	0.99	2.48	3.256 (4)	135
C1*Y*—H1*Y*1⋯O3^vi^	0.99	2.50	3.346 (4)	143

Symmetry codes: (i) 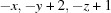; (ii) 
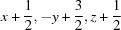
; (iii) 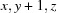; (iv) 
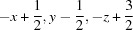
; (v) 
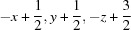
; (vi) 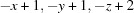.

**Fig. 1.**
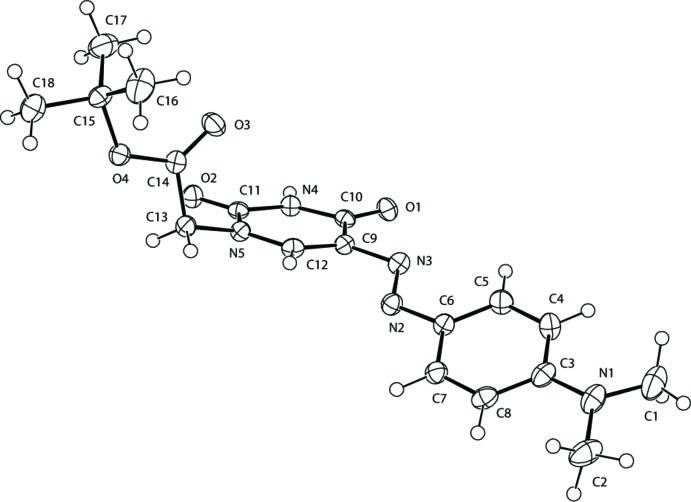
ORTEP representation of the title compound showing the atom-numbering. The asymmetric unit contains an enclathrated CH2Cl2 also.

**Fig. 2.**
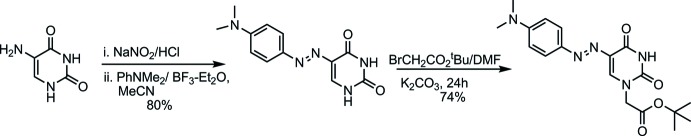
Reaction scheme.

## supplementary crystallographic information

### 1. Introduction

As part of our program in the synthesis of modified nucleobases that possess intrinsic fluorescence while maintaining an unadultered base-paring face, we have prepared an asymmetrical azo compound as a hybrid between a nulceobase and the known fluorescence quencher 4-((4-(di­methyl­amino)­phenyl)­azo)benzoic acid (DABCYL) see: Dodd & Hudson(2009) and Tyagi & Kramer (1996).

### 2. Experimental

The title compound was prepared following standard procedures see: Thurber & Townsend (1972), Tsupak *et al.* (2002) and Moustafa (2011).

### 2.1. Synthesis and crystallization

The title compound was crystallized by slow diffusion of hexanes into a solution of di­chloro­methane. Orange plates of suitable quality for diffraction were obtained.

### 2.2. Refinement

Crystal data, data collection and structure refinement details are summarized in Table 1.

### 3. Results and discussion

The molecule resides at a general position in the lattice as does a disordered methyl­ene chloride of solvation. Selected inter­molecular hydrogen bonding inter­actions are mentioned briefly below. All potential hydrogen bonding inter­actions are listed in Table 1.

The principal inter­molecular inter­actions are N—H···O hydrogen bonds which join uracil moieties across a centre of symmetry forming an eight membered ring, designated as R^2^_2_(8) ring in graph set notation. This hydrogen bond consists of N4—H4A···O2 with an H···A distance of 2.02 (3) Å. The other uracil carbonyl oxygen, O1, participates in two inter­molecular C—H···O hydrogen bonds. The shorter of the two inter­actions arises with the atoms C12—H12 acting as the donor group. The hydrogen bond pattern forms a C(5) chain. The H12···O1 distance is 2.34 (2) Å. The longer inter­action is with the atom H1X1 from the CH_2_Cl_2_ of solvation. The H1X2···O1 distance is 2.48 Å.

### Crystal data

**Table:**

C_18_H_23_N_5_O_4_·CH_2_Cl_2_	*F*(000) = 960
*M**_r_* = 458.34	*D*_x_ = 1.339 Mg m^−^^3^
Monoclinic, *P*2_1_/*n*	Mo *K*α radiation, λ = 0.71073 Å
*a* = 13.208 (9) Å	Cell parameters from 9939 reflections
*b* = 10.783 (6) Å	θ = 2.3–28.1°
*c* = 17.255 (11) Å	µ = 0.32 mm^−^^1^
β = 112.33 (2)°	*T* = 150 K
*V* = 2273 (3) Å^3^	Prism, colourless
*Z* = 4	0.20 × 0.18 × 0.15 mm

### Data collection

**Table:**

Nonius KappaCCD diffractometer	3567 reflections with *I* > 2σ(*I*)
Radiation source: sealed tube	*R*_int_ = 0.054
phi and ω scans	θ_max_ = 29.6°, θ_min_ = 2.3°
Absorption correction: multi-scan (*SADABS*; Bruker, 2008)	*h* = −18→17
*T*_min_ = 0.689, *T*_max_ = 0.746	*k* = −14→14
34970 measured reflections	*l* = −22→23
5944 independent reflections	

### Refinement

**Table:**

Refinement on *F*^2^	Primary atom site location: structure-invariant direct methods
Least-squares matrix: full	Secondary atom site location: difference Fourier map
*R*[*F*^2^ > 2σ(*F*^2^)] = 0.051	Hydrogen site location: inferred from neighbouring sites
*wR*(*F*^2^) = 0.128	H atoms treated by a mixture of independent and constrained refinement
*S* = 1.03	*w* = 1/[σ^2^(*F*_o_^2^) + (0.0446*P*)^2^ + 1.4761*P*] where *P* = (*F*_o_^2^ + 2*F*_c_^2^)/3
5944 reflections	(Δ/σ)_max_ = 0.001
374 parameters	Δρ_max_ = 0.53 e Å^−^^3^
12 restraints	Δρ_min_ = −0.59 e Å^−^^3^

### Special details

**Table:**

Geometry. All e.s.d.'s (except the e.s.d. in the dihedral angle between two l.s. planes) are estimated using the full covariance matrix. The cell e.s.d.'s are taken into account individually in the estimation of e.s.d.'s in distances, angles and torsion angles; correlations between e.s.d.'s in cell parameters are only used when they are defined by crystal symmetry. An approximate (isotropic) treatment of cell e.s.d.'s is used for estimating e.s.d.'s involving l.s. planes.

### Fractional atomic coordinates and isotropic or equivalent isotropic displacement parameters (Å^2^)

**Table:**

	*x*	*y*	*z*	*U*_iso_*/*U*_eq_	Occ. (<1)
C1	0.9052 (2)	0.7150 (3)	1.05913 (18)	0.0474 (7)	
H1A	0.873 (2)	0.777 (2)	1.0869 (16)	0.045 (7)*	
H1B	0.929 (3)	0.766 (3)	1.020 (2)	0.084 (11)*	
H1C	0.969 (3)	0.675 (3)	1.101 (2)	0.083 (11)*	
C2	0.8704 (2)	0.4912 (3)	1.02020 (18)	0.0443 (7)	
H2A	0.939 (3)	0.490 (3)	1.069 (2)	0.071 (9)*	
H2B	0.891 (2)	0.465 (2)	0.9674 (18)	0.056 (8)*	
H2C	0.817 (3)	0.427 (3)	1.0273 (19)	0.071 (10)*	
N1	0.82913 (15)	0.61752 (19)	1.01530 (12)	0.0372 (5)	
C3	0.72814 (16)	0.6469 (2)	0.95471 (13)	0.0279 (5)	
C4	0.68748 (18)	0.7695 (2)	0.94533 (14)	0.0305 (5)	
H4	0.7300 (18)	0.833 (2)	0.9799 (14)	0.028 (6)*	
C5	0.58531 (18)	0.7985 (2)	0.88637 (14)	0.0293 (5)	
H5	0.5580 (19)	0.877 (2)	0.8811 (15)	0.039 (7)*	
C6	0.51979 (16)	0.70655 (18)	0.83333 (12)	0.0232 (4)	
C7	0.56023 (17)	0.58499 (19)	0.84203 (14)	0.0261 (5)	
H7	0.5167 (17)	0.524 (2)	0.8034 (14)	0.029 (6)*	
C8	0.66189 (17)	0.5549 (2)	0.90144 (14)	0.0281 (5)	
H8	0.6874 (18)	0.469 (2)	0.9054 (14)	0.034 (6)*	
N2	0.41469 (13)	0.72338 (16)	0.77085 (10)	0.0250 (4)	
N3	0.37871 (13)	0.83448 (15)	0.76006 (10)	0.0245 (4)	
C9	0.26949 (15)	0.83883 (18)	0.69941 (12)	0.0213 (4)	
C10	0.23176 (16)	0.95536 (18)	0.65479 (12)	0.0210 (4)	
O1	0.28351 (11)	1.05269 (13)	0.66708 (9)	0.0269 (3)	
N4	0.12641 (13)	0.94789 (16)	0.59289 (10)	0.0216 (4)	
H4A	0.1030 (19)	1.012 (2)	0.5641 (16)	0.037 (7)*	
C11	0.05549 (15)	0.84931 (18)	0.57493 (12)	0.0199 (4)	
O2	−0.03756 (11)	0.85176 (13)	0.52046 (8)	0.0254 (3)	
N5	0.09479 (13)	0.74509 (14)	0.62430 (10)	0.0207 (4)	
C12	0.19932 (16)	0.74158 (19)	0.68434 (12)	0.0226 (4)	
H12	0.2182 (18)	0.663 (2)	0.7162 (14)	0.033 (6)*	
C13	0.01573 (17)	0.64735 (19)	0.61964 (14)	0.0229 (4)	
H13A	0.0566 (19)	0.576 (2)	0.6456 (15)	0.036 (6)*	
H13B	−0.0272 (17)	0.6270 (19)	0.5626 (14)	0.023 (5)*	
C14	−0.05649 (16)	0.68765 (18)	0.66563 (12)	0.0220 (4)	
O3	−0.03756 (12)	0.77674 (14)	0.71118 (9)	0.0318 (4)	
O4	−0.14075 (11)	0.60916 (13)	0.64849 (8)	0.0245 (3)	
C15	−0.21469 (16)	0.6153 (2)	0.69607 (13)	0.0271 (5)	
C16	−0.1464 (2)	0.5914 (3)	0.78781 (16)	0.0456 (7)	
H16A	−0.095 (2)	0.659 (3)	0.8133 (19)	0.066 (9)*	
H16B	−0.104 (3)	0.516 (3)	0.7938 (19)	0.070 (10)*	
H16C	−0.193 (3)	0.584 (3)	0.819 (2)	0.072 (10)*	
C17	−0.2744 (2)	0.7388 (3)	0.6801 (2)	0.0440 (6)	
H17A	−0.328 (2)	0.738 (3)	0.7089 (17)	0.057 (8)*	
H17B	−0.311 (2)	0.755 (3)	0.6188 (19)	0.056 (8)*	
H17C	−0.227 (3)	0.807 (3)	0.7051 (19)	0.065 (9)*	
C18	−0.2945 (2)	0.5097 (3)	0.65820 (18)	0.0382 (6)	
H18A	−0.256 (2)	0.432 (2)	0.6654 (15)	0.040 (7)*	
H18B	−0.337 (2)	0.524 (3)	0.595 (2)	0.067 (9)*	
H18C	−0.347 (2)	0.506 (3)	0.6847 (17)	0.054 (8)*	
C1X	0.8190 (3)	0.1657 (3)	1.1140 (2)	0.0728 (10)	0.630 (13)
H1X1	0.8564	0.1666	1.1758	0.087*	0.630 (13)
H1X2	0.7807	0.2460	1.0970	0.087*	0.630 (13)
Cl1X	0.91767 (6)	0.15439 (9)	1.07048 (5)	0.0687 (3)	0.630 (13)
Cl2X	0.72201 (17)	0.0480 (2)	1.08651 (16)	0.0511 (7)	0.630 (13)
C1Y	0.8190 (3)	0.1657 (3)	1.1140 (2)	0.0728 (10)	0.370 (13)
H1Y1	0.8550	0.1888	1.1739	0.087*	0.370 (13)
H1Y2	0.7669	0.2326	1.0853	0.087*	0.370 (13)
Cl1Y	0.91767 (6)	0.15439 (9)	1.07048 (5)	0.0687 (3)	0.370 (13)
Cl2Y	0.7489 (11)	0.0313 (6)	1.1063 (8)	0.114 (2)	0.370 (13)

### Atomic displacement parameters (Å^2^)

**Table:**

	*U*^11^	*U*^22^	*U*^33^	*U*^12^	*U*^13^	*U*^23^
C1	0.0272 (13)	0.072 (2)	0.0370 (15)	−0.0029 (13)	0.0051 (12)	0.0058 (15)
C2	0.0346 (14)	0.0589 (18)	0.0386 (15)	0.0208 (13)	0.0128 (12)	0.0140 (13)
N1	0.0243 (9)	0.0461 (12)	0.0349 (11)	0.0039 (9)	0.0042 (8)	0.0078 (9)
C3	0.0231 (10)	0.0355 (12)	0.0267 (11)	0.0020 (9)	0.0112 (9)	0.0078 (9)
C4	0.0291 (11)	0.0286 (12)	0.0293 (11)	−0.0049 (10)	0.0061 (10)	−0.0004 (10)
C5	0.0305 (11)	0.0224 (11)	0.0322 (12)	0.0019 (9)	0.0087 (10)	0.0025 (9)
C6	0.0223 (10)	0.0211 (10)	0.0257 (10)	0.0003 (8)	0.0083 (8)	0.0028 (8)
C7	0.0241 (10)	0.0226 (11)	0.0331 (12)	−0.0018 (9)	0.0126 (9)	0.0001 (9)
C8	0.0274 (11)	0.0238 (11)	0.0362 (12)	0.0046 (9)	0.0154 (10)	0.0069 (9)
N2	0.0231 (9)	0.0244 (9)	0.0264 (9)	0.0010 (7)	0.0083 (7)	0.0023 (7)
N3	0.0241 (9)	0.0250 (9)	0.0238 (9)	0.0010 (7)	0.0085 (7)	0.0040 (7)
C9	0.0211 (9)	0.0227 (10)	0.0199 (10)	0.0007 (8)	0.0076 (8)	0.0011 (8)
C10	0.0250 (10)	0.0207 (10)	0.0196 (9)	0.0000 (8)	0.0109 (8)	−0.0008 (8)
O1	0.0308 (8)	0.0201 (7)	0.0284 (8)	−0.0036 (6)	0.0097 (6)	−0.0010 (6)
N4	0.0235 (8)	0.0181 (9)	0.0221 (9)	0.0009 (7)	0.0073 (7)	0.0038 (7)
C11	0.0233 (10)	0.0190 (10)	0.0206 (10)	0.0012 (8)	0.0117 (8)	0.0008 (8)
O2	0.0236 (7)	0.0233 (7)	0.0257 (7)	0.0011 (6)	0.0052 (6)	0.0032 (6)
N5	0.0211 (8)	0.0177 (8)	0.0237 (8)	−0.0002 (7)	0.0088 (7)	0.0026 (7)
C12	0.0251 (10)	0.0207 (11)	0.0226 (10)	0.0040 (8)	0.0096 (9)	0.0038 (8)
C13	0.0229 (10)	0.0170 (10)	0.0287 (11)	−0.0010 (8)	0.0097 (9)	0.0017 (9)
C14	0.0228 (10)	0.0193 (10)	0.0224 (10)	−0.0006 (8)	0.0068 (8)	0.0053 (8)
O3	0.0373 (9)	0.0270 (8)	0.0348 (8)	−0.0071 (7)	0.0179 (7)	−0.0087 (7)
O4	0.0248 (7)	0.0231 (7)	0.0287 (8)	−0.0038 (6)	0.0138 (6)	−0.0004 (6)
C15	0.0244 (10)	0.0315 (12)	0.0297 (11)	−0.0011 (9)	0.0151 (9)	0.0013 (9)
C16	0.0399 (15)	0.069 (2)	0.0298 (13)	−0.0125 (15)	0.0155 (12)	0.0091 (13)
C17	0.0367 (14)	0.0416 (16)	0.0602 (19)	0.0039 (12)	0.0255 (14)	−0.0042 (14)
C18	0.0325 (13)	0.0400 (15)	0.0457 (15)	−0.0086 (11)	0.0189 (12)	−0.0017 (12)
C1X	0.085 (2)	0.0521 (19)	0.105 (3)	−0.0259 (17)	0.063 (2)	−0.0354 (18)
Cl1X	0.0585 (5)	0.0981 (7)	0.0553 (5)	−0.0121 (4)	0.0282 (4)	−0.0193 (4)
Cl2X	0.0499 (14)	0.0513 (13)	0.0461 (11)	−0.0163 (7)	0.0114 (11)	−0.0014 (7)
C1Y	0.085 (2)	0.0521 (19)	0.105 (3)	−0.0259 (17)	0.063 (2)	−0.0354 (18)
Cl1Y	0.0585 (5)	0.0981 (7)	0.0553 (5)	−0.0121 (4)	0.0282 (4)	−0.0193 (4)
Cl2Y	0.232 (6)	0.0284 (17)	0.153 (5)	−0.024 (3)	0.155 (5)	−0.013 (3)

### Geometric parameters (Å, º)

**Table:**

C1—N1	1.452 (3)	C11—O2	1.232 (2)
C1—H1A	1.00 (3)	C11—N5	1.386 (2)
C1—H1B	1.01 (4)	N5—C12	1.376 (3)
C1—H1C	0.98 (4)	N5—C13	1.464 (3)
C2—N1	1.458 (3)	C12—H12	0.99 (2)
C2—H2A	0.98 (3)	C13—C14	1.518 (3)
C2—H2B	1.08 (3)	C13—H13A	0.95 (2)
C2—H2C	1.03 (3)	C13—H13B	0.96 (2)
N1—C3	1.383 (3)	C14—O3	1.206 (2)
C3—C8	1.408 (3)	C14—O4	1.339 (2)
C3—C4	1.414 (3)	O4—C15	1.497 (3)
C4—C5	1.382 (3)	C15—C16	1.517 (3)
C4—H4	0.94 (2)	C15—C17	1.518 (3)
C5—C6	1.403 (3)	C15—C18	1.521 (3)
C5—H5	0.91 (3)	C16—H16A	0.98 (3)
C6—C7	1.402 (3)	C16—H16B	0.97 (3)
C6—N2	1.409 (3)	C16—H16C	0.95 (3)
C7—C8	1.383 (3)	C17—H17A	1.00 (3)
C7—H7	0.95 (2)	C17—H17B	1.00 (3)
C8—H8	0.98 (2)	C17—H17C	0.96 (3)
N2—N3	1.276 (2)	C18—H18A	0.97 (3)
N3—C9	1.425 (3)	C18—H18B	1.03 (3)
C9—C12	1.358 (3)	C18—H18C	0.97 (3)
C9—C10	1.459 (3)	C1X—Cl2X	1.737 (3)
C10—O1	1.226 (2)	C1X—Cl1X	1.738 (3)
C10—N4	1.398 (3)	C1X—H1X1	0.9900
N4—C11	1.372 (3)	C1X—H1X2	0.9900
N4—H4A	0.84 (3)		
			
N1—C1—H1A	113.5 (15)	C12—N5—C11	121.27 (16)
N1—C1—H1B	112.0 (19)	C12—N5—C13	120.81 (16)
H1A—C1—H1B	104 (2)	C11—N5—C13	117.19 (16)
N1—C1—H1C	108 (2)	C9—C12—N5	122.66 (18)
H1A—C1—H1C	110 (2)	C9—C12—H12	123.2 (13)
H1B—C1—H1C	109 (3)	N5—C12—H12	114.2 (13)
N1—C2—H2A	105.4 (19)	N5—C13—C14	110.01 (17)
N1—C2—H2B	113.6 (14)	N5—C13—H13A	107.0 (14)
H2A—C2—H2B	106 (2)	C14—C13—H13A	110.0 (14)
N1—C2—H2C	112.1 (17)	N5—C13—H13B	110.7 (13)
H2A—C2—H2C	110 (2)	C14—C13—H13B	111.1 (13)
H2B—C2—H2C	109 (2)	H13A—C13—H13B	107.9 (19)
C3—N1—C1	120.4 (2)	O3—C14—O4	126.64 (19)
C3—N1—C2	119.3 (2)	O3—C14—C13	123.57 (18)
C1—N1—C2	118.5 (2)	O4—C14—C13	109.78 (17)
N1—C3—C8	121.0 (2)	C14—O4—C15	120.76 (16)
N1—C3—C4	121.1 (2)	O4—C15—C16	108.16 (19)
C8—C3—C4	117.98 (19)	O4—C15—C17	109.97 (18)
C5—C4—C3	121.2 (2)	C16—C15—C17	113.6 (2)
C5—C4—H4	118.8 (13)	O4—C15—C18	102.75 (18)
C3—C4—H4	120.0 (13)	C16—C15—C18	111.2 (2)
C4—C5—C6	120.6 (2)	C17—C15—C18	110.6 (2)
C4—C5—H5	121.4 (15)	C15—C16—H16A	112.1 (18)
C6—C5—H5	118.0 (15)	C15—C16—H16B	110.1 (19)
C7—C6—C5	118.39 (19)	H16A—C16—H16B	108 (2)
C7—C6—N2	115.27 (18)	C15—C16—H16C	109.9 (19)
C5—C6—N2	126.34 (19)	H16A—C16—H16C	107 (3)
C8—C7—C6	121.4 (2)	H16B—C16—H16C	110 (3)
C8—C7—H7	121.1 (13)	C15—C17—H17A	108.7 (16)
C6—C7—H7	117.4 (13)	C15—C17—H17B	110.5 (17)
C7—C8—C3	120.4 (2)	H17A—C17—H17B	112 (2)
C7—C8—H8	119.0 (13)	C15—C17—H17C	112.6 (18)
C3—C8—H8	120.6 (13)	H17A—C17—H17C	104 (2)
N3—N2—C6	115.89 (16)	H17B—C17—H17C	108 (2)
N2—N3—C9	110.80 (16)	C15—C18—H18A	110.5 (15)
C12—C9—N3	122.83 (18)	C15—C18—H18B	110.8 (16)
C12—C9—C10	119.52 (18)	H18A—C18—H18B	109 (2)
N3—C9—C10	117.61 (17)	C15—C18—H18C	109.2 (17)
O1—C10—N4	120.61 (18)	H18A—C18—H18C	110 (2)
O1—C10—C9	126.13 (18)	H18B—C18—H18C	107 (2)
N4—C10—C9	113.26 (17)	Cl2X—C1X—Cl1X	115.3 (2)
C11—N4—C10	127.82 (17)	Cl2X—C1X—H1X1	108.5
C11—N4—H4A	116.1 (17)	Cl1X—C1X—H1X1	108.5
C10—N4—H4A	116.0 (17)	Cl2X—C1X—H1X2	108.5
O2—C11—N4	123.60 (18)	Cl1X—C1X—H1X2	108.5
O2—C11—N5	121.23 (17)	H1X1—C1X—H1X2	107.5
N4—C11—N5	115.15 (17)		
			
C1—N1—C3—C8	168.5 (2)	N3—C9—C10—N4	175.99 (16)
C2—N1—C3—C8	4.1 (3)	O1—C10—N4—C11	−175.49 (18)
C1—N1—C3—C4	−12.6 (3)	C9—C10—N4—C11	4.9 (3)
C2—N1—C3—C4	−177.0 (2)	C10—N4—C11—O2	177.93 (19)
N1—C3—C4—C5	−178.2 (2)	C10—N4—C11—N5	−0.6 (3)
C8—C3—C4—C5	0.7 (3)	O2—C11—N5—C12	179.03 (18)
C3—C4—C5—C6	−0.9 (3)	N4—C11—N5—C12	−2.4 (3)
C4—C5—C6—C7	0.4 (3)	O2—C11—N5—C13	−10.7 (3)
C4—C5—C6—N2	−180.0 (2)	N4—C11—N5—C13	167.88 (17)
C5—C6—C7—C8	0.3 (3)	N3—C9—C12—N5	−178.38 (18)
N2—C6—C7—C8	−179.39 (19)	C10—C9—C12—N5	4.2 (3)
C6—C7—C8—C3	−0.5 (3)	C11—N5—C12—C9	0.5 (3)
N1—C3—C8—C7	178.9 (2)	C13—N5—C12—C9	−169.43 (19)
C4—C3—C8—C7	0.0 (3)	C12—N5—C13—C14	96.3 (2)
C7—C6—N2—N3	−177.35 (18)	C11—N5—C13—C14	−74.0 (2)
C5—C6—N2—N3	3.0 (3)	N5—C13—C14—O3	−12.2 (3)
C6—N2—N3—C9	−176.25 (16)	N5—C13—C14—O4	168.70 (15)
N2—N3—C9—C12	26.9 (3)	O3—C14—O4—C15	−8.9 (3)
N2—N3—C9—C10	−155.63 (17)	C13—C14—O4—C15	170.22 (16)
C12—C9—C10—O1	173.92 (19)	C14—O4—C15—C16	−60.9 (2)
N3—C9—C10—O1	−3.6 (3)	C14—O4—C15—C17	63.7 (2)
C12—C9—C10—N4	−6.5 (3)	C14—O4—C15—C18	−178.54 (18)

### Hydrogen-bond geometry (Å, º)

**Table:**

*D*—H···*A*	*D*—H	H···*A*	*D*···*A*	*D*—H···*A*
N4—H4*A*···O2^i^	0.84 (3)	2.02 (3)	2.851 (2)	171 (2)
C2—H2*C*···O1^ii^	1.03 (3)	2.62 (3)	3.189 (4)	115 (2)
C4—H4···Cl2*X*^iii^	0.94 (2)	2.98 (2)	3.784 (4)	143.5 (17)
C12—H12···O1^iv^	0.99 (2)	2.34 (2)	3.214 (3)	145.8 (18)
C13—H13*B*···Cl2*X*^v^	0.96 (2)	2.99 (2)	3.895 (4)	158.6 (16)
C16—H16*A*···O3	0.98 (3)	2.51 (3)	3.042 (3)	114 (2)
C17—H17*C*···O3	0.96 (3)	2.48 (3)	2.994 (4)	113 (2)
C1*X*—H1*X*1···O3^vi^	0.99	2.52	3.346 (4)	141
C1*X*—H1*X*2···O1^ii^	0.99	2.48	3.256 (4)	135
C1*Y*—H1*Y*1···O3^vi^	0.99	2.50	3.346 (4)	143

Symmetry codes: (i) −*x*, −*y*+2, −*z*+1; (ii) *x*+1/2, −*y*+3/2, *z*+1/2; (iii) *x*, *y*+1, *z*; (iv) −*x*+1/2, *y*−1/2, −*z*+3/2; (v) −*x*+1/2, *y*+1/2, −*z*+3/2; (vi) −*x*+1, −*y*+1, −*z*+2.

## References

[bb1] Asanuma, H., Ito, T., Yoshida, T., Liang, X. & Komiyama, M. (1999). *Angew. Chem. Int. Ed. Engl.* **38**, 2393–2395. 10.1002/(sici)1521-3773(19990816)38:16<2393::aid-anie2393>3.0.co;2-7 10458798

[bb13] Boyle, P. D. (2008). http://www.xray.ncsu.edu/PyCIFUtils/

[bb2] Bruker (2008). *SAINT* Bruker AXS Inc., Madison, Wisconsin, USA.

[bb3] Dodd, D. W. & Hudson, R. H. E. (2009). *Mini Rev. Org. Chem.* **4**, 378–39.

[bb4] Gabe, E. J., Le Page, Y., Charland, J.-P., Lee, F. L. & White, P. S. (1989). *J. Appl. Cryst.* **22**, 384–387.

[bb5] Moustafa, M. E. (2011). Ph.D. thesis, The University of Western Ontario, London, Ontario, Canada.

[bb6] Moustafa, M. E. & Hudson, R. H. E. (2011). *Nucleosides Nucleotides Nucleic Acids* **30**, 740–751. 10.1080/15257770.2011.604661 21902475

[bb7] Nonius (1999). *COLLECT* Nonius BV, Delft, The Netherlands.

[bb8] Sheldrick, G. M. (2008). *Acta Cryst.* A**64**, 112–122. 10.1107/S0108767307043930 18156677

[bb9] Thurber, T. C. & Townsend, L. B. (1972). *J. Heterocycl. Chem.* **9**, 629–636.

[bb10] Tsupak, E. B., Shevchenko, M. A., Tkachenko, Y. N. & Nazarov, D. A. (2002). *Russ. J. Org. Chem.* **38**, 923–930.

[bb11] Tyagi, S. & Kramer, F. R. (1996). *Nat. Biotechnol.* **14**, 303–308. 10.1038/nbt0396-303 9630890

[bb12] Yue, S. S., Li, J. D., Zhang, J. Y., Lu, J. J. & Chen, M. (2009). *Chin. Chem. Bull.* **54**, 4753–4755.

